# Atrial tachycardia originating from a right atrial free wall diverticulum: case report

**DOI:** 10.1093/ehjcr/ytae497

**Published:** 2024-09-12

**Authors:** Shuang Zhang, Yichao Xiao, Die Hu, Mingxian Chen, Xuping Li

**Affiliations:** Department of Cardiovascular Medicine, The Second Xiangya Hospital of Central South University, 139 Renmin Road, Changsha City, Hunan Province 410000, China; Department of Cardiovascular Medicine, The Second Xiangya Hospital of Central South University, 139 Renmin Road, Changsha City, Hunan Province 410000, China; Department of Cardiovascular Medicine, The Second Xiangya Hospital of Central South University, 139 Renmin Road, Changsha City, Hunan Province 410000, China; Department of Cardiovascular Medicine, The Second Xiangya Hospital of Central South University, 139 Renmin Road, Changsha City, Hunan Province 410000, China; Department of Cardiovascular Medicine, The Second Xiangya Hospital of Central South University, 139 Renmin Road, Changsha City, Hunan Province 410000, China

**Keywords:** Arrhythmia, Ablation, Atrial tachycardia, Diverticulum, Case report

## Abstract

**Background:**

Atrial tachycardia (AT) is an arrhythmic disorder originating from the atrium, independent of the atrioventricular node, and includes various types based on different mechanisms such as abnormal automaticity, triggered activity, and re-entry. These mechanisms are often related to specific anatomical structures. Focal AT, though relatively rare, typically arises from well-known locations in the left and right atria, such as the pulmonary veins, mitral valve annulus, crista terminalis, and coronary sinus ostium.

**Case summary:**

We report a rare case of AT originating from a diverticulum in the right atrial free wall. The patient experienced recurrent AT episodes resistant to standard treatments. Detailed electrophysiological mapping identified the unusual origin of the tachycardia from a right atrial free wall diverticulum. Catheter ablation was successfully performed, leading to the resolution of the arrhythmia, with the patient remaining symptom-free during follow-up.

**Discussion:**

This case expands the understanding of AT origins, highlighting the right atrial free wall diverticulum as a potential, though rare, source of tachycardia. The case emphasizes the importance of comprehensive electrophysiological mapping, especially in atypical presentations of AT. Successful ablation in this instance underscores the potential for targeted interventions even in uncommon anatomical sites. Further studies are needed to assess the prevalence and clinical significance of AT arising from such rare locations.

**Learning points**

- Anatomical variations and electrophysiology:
  - Highlights the importance of understanding the intricate relationship between cardiac anatomy and electrophysiological mechanisms.
  - Identifies an atrial tachycardia originating from a right atrial free wall diverticulum.
  - Emphasizes the need for comprehensive pre-procedural imaging and mapping techniques.
  - Stresses the significance of integrating anatomical knowledge with electrophysiological principles in diagnosing and managing cardiac arrhythmias.
- Multidisciplinary approach to diagnosis and treatment:
  - Management of complex arrhythmias often necessitates a multidisciplinary approach.
  - Collaboration between electrophysiologists, imaging specialists, and cardiac surgeons is crucial.
  - Successful diagnosis and treatment required close cooperation to accurately localize the arrhythmogenic focus.
  - Planning the ablation procedure and ensuring optimal patient outcomes relied on inter-disciplinary teamwork and expertize.
  - Highlights the importance of such teamwork in navigating challenging cases of cardiac arrhythmias.

## Introduction

Atrial tachycardia (AT) is a common arrhythmic disorder. It encompasses a range of tachycardia types originating from the atrium, whose sustenance does not depend on the atrioventricular node. These various arrhythmias have different mechanisms and are often associated with specific anatomical structures, including abnormal automaticity, triggered activities, and re-entry phenomena. Current research has classified conventional AT based on electrophysiological mechanisms and anatomical structures into either focal or macro re-entrant types.^1^

Focal AT is a relatively rare arrhythmic disorder, which has specific anatomical distributions,^2^ related to changes in the direction of myocardial fibres or the junctions of two different tissues. In the left atrium, most originate from the pulmonary veins, mitral valve annulus, left atrial appendage, and left atrial septum. In the right atrium, such tachycardias tend to occur at the crista terminalis, coronary sinus ostium, tricuspid valve annulus, right atrial appendage, and right atrial septum. We report a rare case of AT originating from a diverticulum in the right atrial free wall.

## Summary figure

The diverticulum displayed a grey colour with no electrical potential. The earliest site of activation was at the opening of the diverticulum, which appeared white in colour, followed by the spread of activation towards the atria.

**Figure:**
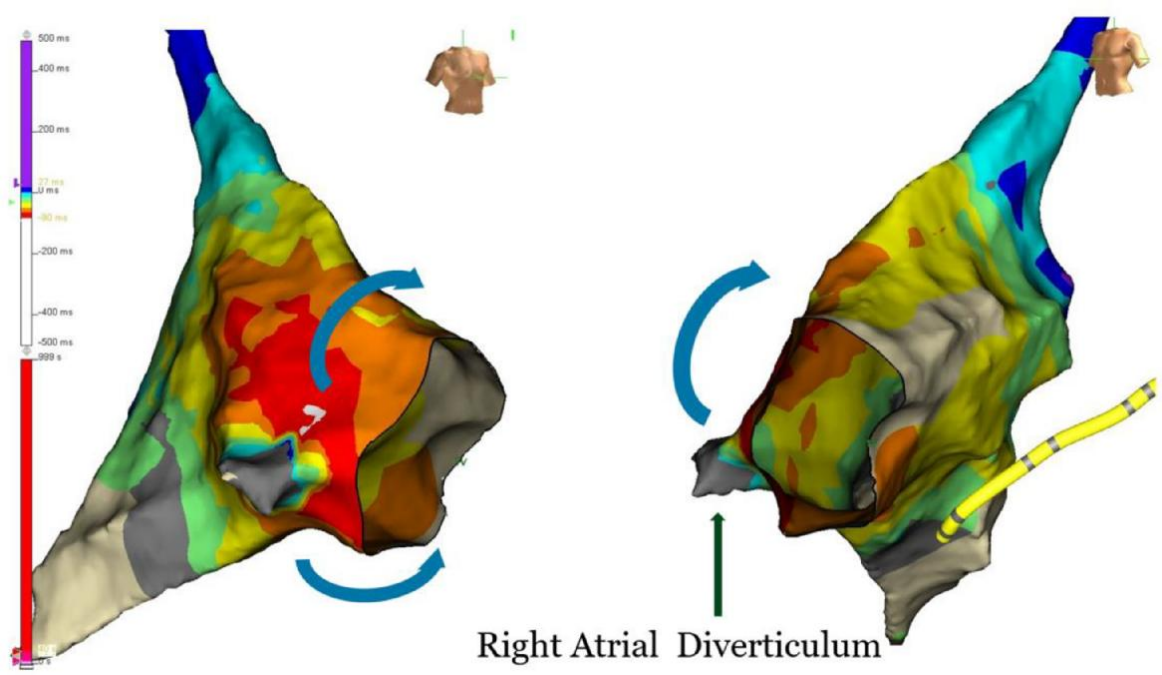


## Case presentation

**Figure 1:**
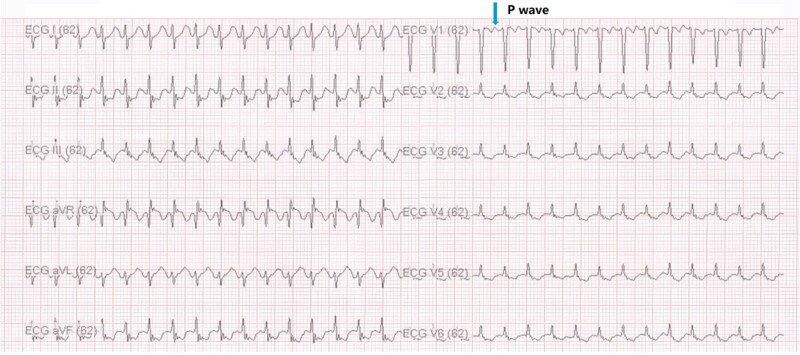
The 12-lead electrocardiogram (ECG) of the patient during tachycardia. The *P* waves are positive in leads I, avL, and V1-V6, and negative in leads II, III, avF, and avR, suggesting that the origin of the atrial tachycardia is from the lower edge of the right atrial free wall.

The patient, a 35-year-old female, was admitted to the hospital on 25 August 2023, for paroxysmal palpitations that she had experienced for 5 years. The patient was treated with beta-blockers during this period, but there was no significant improvement. The palpitations occurred intermittently, with the longest duration reaching up to 30 min. Holter monitoring indicated 8743 atrial premature beats and 160 episodes of AT within 24 h. Echocardiography showed normal cardiac structures (ejection fraction: 68%). Chest X-ray examination showed no obvious abnormalities. During an arrhythmia episode, the electrocardiogram (ECG) during tachycardia is shown in *Figure 1*. The patient had no significant past medical history. Based on the examination results, the current diagnosis is ‘Atrial Tachycardia, AT’.

**Figure 2:**
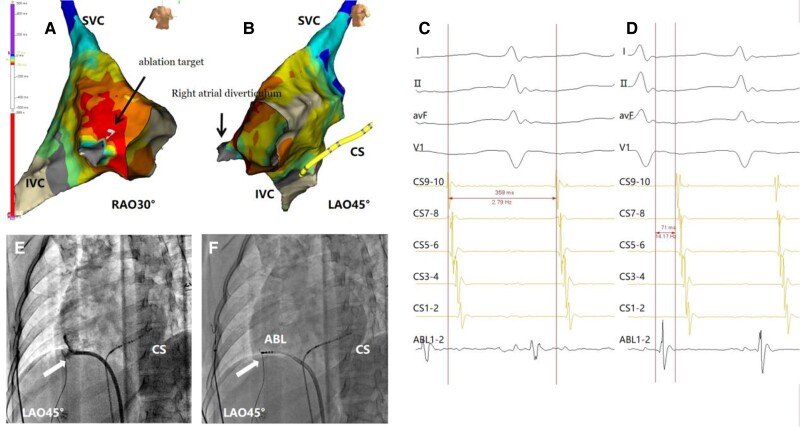
(*A*) The 3D electroanatomical model in right anterior oblique (RAO) 30° view shows activation from white to purple, with grey indicating no potential. Activation starts from the diverticulum ostium (black arrow) towards the atrium. The diverticulum interior is grey, indicating no potential. (*B*) The 3D model in left anterior oblique (LAO) 45° view shows the right atrial diverticulum (black arrow). (*C*) Tachycardia cycle length is 359 ms. (*D*) The earliest A-wave recorded at the diverticulum opening leads the proximal coronary sinus electrode (CSp) by 71 ms. (*E*) X-ray in LAO 45° view after retrograde angiography shows the diverticulum (white arrow). (*F*) X-ray in LAO 45° view shows the ablation catheter's target at the diverticulum orifice (white arrow; ABL, ablation; CS, coronary sinus electrode; SVC, superior vena cava; IVC, inferior vena cava).

During the intra-cardiac electrophysiological study, atrial premature beats originating from the free wall of the right atrium (RA) can easily induce tachycardia, with a tachycardia cycle length of 319 ms (*Figure 2C*). During tachycardia, ventricular S1S1 pacing at 300 ms resulted in a V-A-A-V sequence on the first beat post-pacing. The electrophysiological diagnosis confirmed AT. Activation mapping of the RA was performed using a three-dimensional (3D) mapping catheter (Advisor HD Grid, Abbott) and confirmed that the AT was a focal AT. Under 3D mapping, the earliest A-wave was located at the 8 o’clock direction on the right atrial free wall. Subsequently, detailed mapping was performed using an ablation catheter. Further detailed mapping revealed that the ablation catheter could be inserted into a ‘pouch,’ with an observed increase in impedance, and a diverticulum on the right atrial free wall was visible in the 3D model (*Figure 2B*). After the catheter entered the diverticulum, the impedance increased from 125 to 170 Ω. Electrophysiological mapping and detailed measurement showed no potential within the diverticulum (*Figure 2A*). The earliest A-wave was recorded at the orifice of the diverticulum, leading the proximal coronary sinus electrode (CSp) by 71 ms (*Figure 2A, 2D*). Angiography was performed through the right femoral vein sheath (*Figure 2E*), and a left anterior oblique (LAO) 45° view revealed a protrusion on the right atrial free wall.

After discovering the free wall diverticulum and confirming that the AT was related to the diverticulum, we consulted with cardiac surgeons. We confirmed that the arrhythmia originated not from the deep part of the diverticulum but from its opening. The cardiac surgeons reviewed the patient’s medical records and angiographic images and determined that the patient’s diverticulum was small and we thought that ablation would not be very difficult. Considering our centre’s experience with diverticulum-related ablation, they suggested proceeding with ablation first. If the ablation was unsuccessful, surgical removal would be considered.

The ablation target was combined with the same position LAO 45° X-ray image, locating the ablation catheter at the ostium of the diverticulum (*Figure 2F*). Ablation at this site terminated the AT, and tachycardia could not be induced again.

In the ablation procedure, considering the thin wall of the right atrial free wall diverticulum, excessive ablation carries the risk of perforation or pericardial effusion. The catheter should not be advanced into the diverticulum, and impedance changes should be closely monitored. If the impedance changes by more than 20 Ω, indicating entry into the diverticulum, ablation should be stopped, and the catheter should be withdrawn. Additionally, catheter pressure should be maintained between 5 and 10 g to avoid the risk of perforation. A saline-irrigated ablation catheter (35W, 43°C, 17 ml/min) should be used, with ablation time controlled by the lesion side index (LSI) value. The LSI value should be maintained between 3.0 and 3.5 and not exceed 4.0 to avoid cardiac tamponade.

**Figure 3:**
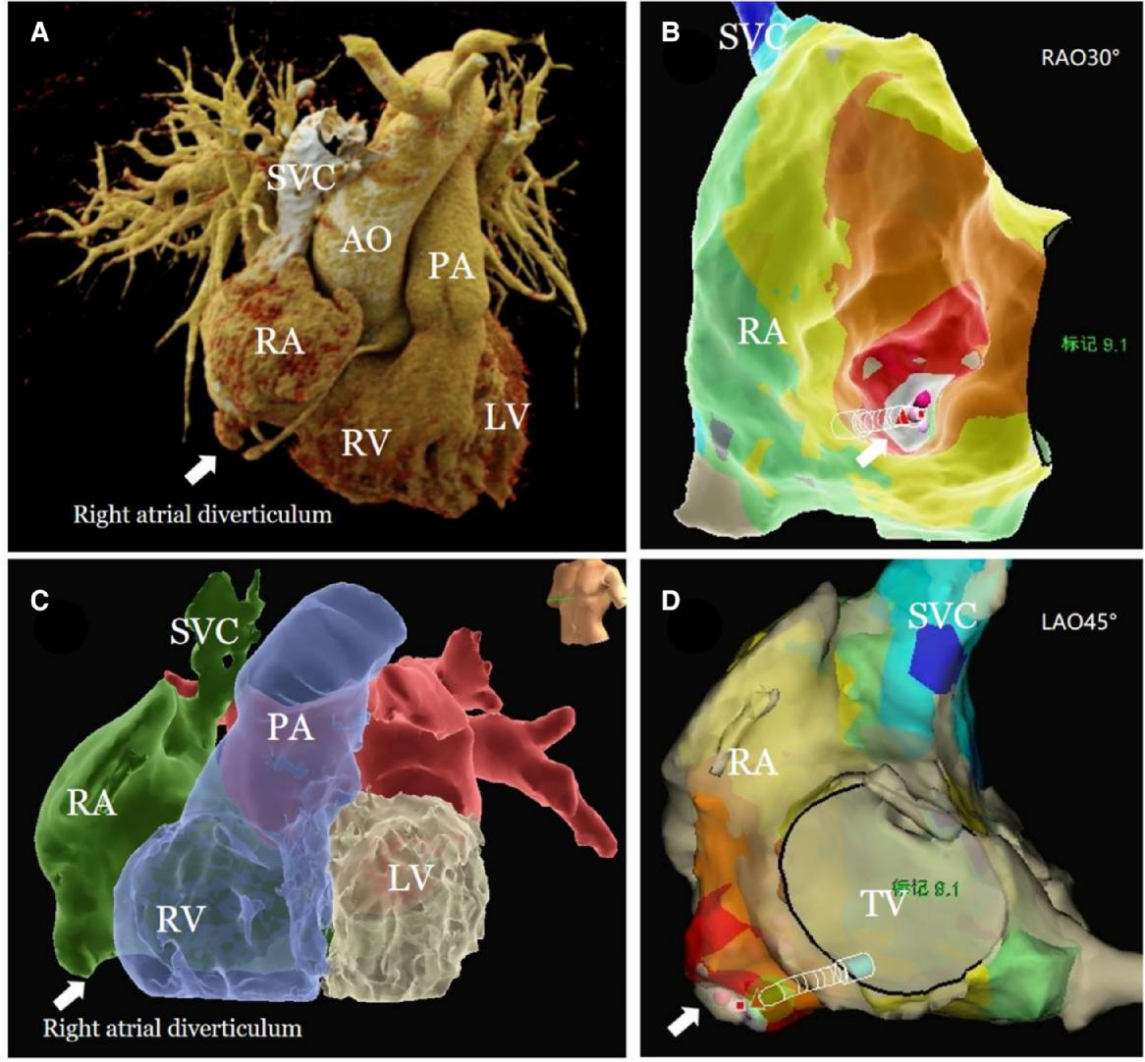
(*A*) Cardiac computed tomography angiography (CTA) with 3D reconstruction shows a right atrial diverticulum on the lower free wall (white arrow). (*B*) Fused cardiac CTA and electroanatomical model in right anterior oblique 30° view indicates the diverticulum (white arrow). (*C*) CTA imported into Ensite system shows the diverticulum at the same location as in (*A*). (*D*) Fused CTA and electroanatomical model in left anterior oblique 45° view highlights the diverticulum (white arrow).

Postoperatively, the patient underwent a right atrial computed tomography angiography (CTA) examination (*Figure 3A*), which further confirmed the presence of a right atrial diverticulum. The CTA images were imported into the Ensite system for model reconstruction (*Figure 3C*), and the CTA reconstruction model was then fused with the electroanatomical reconstruction model (*Figure 3B, 3D*). This demonstrated that the diverticulum identified in both the CTA and electroanatomical reconstructions was located in the same area.

## Follow-up

During the 6-month follow-up period, phone follow-ups were conducted every 2 months. If the patient experienced palpitations, an immediate ECG would be performed to check for the recurrence of AT, indicating ablation failure. In the absence of palpitations, a 24-h Holter monitor was conducted every 3 months. If the Holter monitor indicated AT lasting more than 30 s with ECG characteristics consistent with pre-ablation episodes, it suggested surgical failure. If the ablation was unsuccessful, the patient was advised to consult with a cardiac surgeon for potential surgical removal of the diverticulum. During the 6-month follow-up, the patient experienced no recurrence.

## Discussion

Arrhythmias are often closely related to anatomical abnormalities and the junctions between different tissues, and the occurrence of focal AT is similarly associated with these factors. The presence of a diverticulum can directly stimulate the heart, manifesting as AT. Additionally, the unique fibre orientation and abnormal surface area of the diverticulum provide a substrate for the maintenance of re-entrant AT.^3^ The arrhythmia reported in this case is closely associated with a right atrial diverticulum, as demonstrated by our electroanatomic 3D reconstruction and CTA models.

Literature on right atrial diverticula is currently sparse, with their origins possibly classified into congenital and secondary causes.^3–5^ The aetiology of congenital right atrial diverticula remains unclear, but they may be associated with other congenital heart anomalies, including hypertrophic cardiomyopathy, ventricular septal defects, persistent left superior vena cava, as well as abnormalities in other systems such as bronchial system malformations, hepatic adenomas, and dilation of the renal pelvis and ureteral system.^5^ Secondary factors for the formation of right atrial diverticula are indicated to be related to rheumatic diseases. Literature reports^6^ a case of re-entrant AT caused by a right atrial diverticulum associated with rheumatic disease. In this case, the presence of the diverticulum was confirmed by ultrasound, angiography, and CTA. The patient returned to sinus rhythm after the surgical excision of the diverticulum.^6^

Arrhythmias associated with right atrial diverticula are commonly seen as atrioventricular re-entrant tachycardia,^7,8^ AT,^9^ atrial flutter,^3^ and atrial fibrillation.^10–12^ In all cases, the presence of the diverticulum was confirmed by 3D mapping, echocardiography, and CTA. The 3D system mapping confirmed that the arrhythmias originated from the diverticulum. Ablation was successfully performed at the junction between the diverticulum and the atrium in five cases. In two cases, considering the significant size of the right atrial diverticula, ablation was challenging, and sinus rhythm was maintained with oral medication following electrical cardioversion.

Literature reports indicate that enlarged right atria and diverticulum walls often exhibit lipomatous degeneration and a reduction in myocardial components.^5^ Studies involving pathological examination of tissues excised from right atrial diverticula have shown that these diverticula lack myocardial tissue and are characterized by fibrous connective tissue structures.^13^ The heterogeneity of such tissue provides a substrate for arrhythmias. In this case, 3D electroanatomical mapping and right atrial CTA confirmed the presence of an abnormal structure in the direction of the lower right atrial free wall. The catheter’s penetration into this structure did not yield any electrophysiological recordings, suggesting the absence of significant myocardial structure. This further confirms that junctional and variant tissues serve as substrates for arrhythmogenesis.

Relevant literature indicates that for cardiac diverticula that are large, causing left ventricular compression symptoms, or presenting a risk of thromboembolism, surgical resection should be considered first.^14^ In this case, the patient had a right atrial diverticulum, which was small. After a consultation and discussion with cardiac surgeons, it was determined that there were no compression symptoms or embolization risks. Therefore, radiofrequency ablation was recommended as the initial treatment to terminate the tachycardia, with surgical resection to be considered if ablation was unsuccessful. Furthermore, for patients with normal cardiac function, routine cardiac imaging (such as computed tomography scans) is usually not performed before intra-cardiac electrophysiological examinations. Therefore, the 3D electroanatomical reconstruction system plays a crucial role in identifying arrhythmias originating from diverticula during surgery. It is essential to meticulously map any abnormalities in the right atrial anatomy during the procedure. If a significant anomaly is present, right atrial angiography can be conducted for further confirmation. Electrophysiological mapping can then definitively determine if the arrhythmia originates from a diverticulum.

In the ablation procedure, care should be taken not to advance the catheter into the diverticulum to avoid cardiac perforation. Monitor impedance changes closely; if the impedance increases by more than 20 Ω, proceed with caution, stop ablation, and withdraw the catheter. Maintain stable ablation pressure, keeping it below 10 g to avoid perforation risk. Additionally, ensure that the AI value for saline-irrigated catheter ablation does not exceed 4.0 to prevent pericardial tamponade.

## Lead author biography


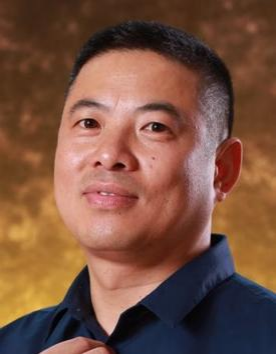


Li Xuping, Professor, Xiangya Second Hospital of Central South University, Young Committee Member of the Chinese Society of Pacing and Electrophysiology, Young Committee Member of the Chinese Biomedical Engineering Society, National Committee Member of the Chinese Society of Electrocardiology, Chairman of the Pacing and Electrophysiology Society of Hunan Medical Association. Visiting Scholar in Germany at Hamburg for 6 months in 2012 and in the United States at Mayo Clinic for 1 year in 2015. Participated in or led six national or provincial-level funded projects, and received three second prizes for national and provincial scientific and technological progress. Published over 16 SCI papers as the first or corresponding author.

## Data Availability

The data underlying this article will be shared on reasonable request to the corresponding author.

## Notes

**Consent:** We confirm that informed consent was obtained from the patient in compliance with the Committee on Publication Ethics (COPE) guidelines. The patient was fully informed about the nature of the case report and agreed to the use of their clinical data and images for publication purposes. The patient’s identity has been protected, and no identifiable information has been disclosed.

**Funding:** Financial support was obtained from the National Natural Science Foundation of China (No. 82150006) and Hunan Provincial Natural Science Foundation (No. 2019JJ40433).
